# Effects of propolis extract supplementation in breeder and broiler diets and it's in ovo injection on immune status, blood parameters, vaccine-antibody response and intestinal microflora of broiler chick

**DOI:** 10.1007/s11250-025-04329-3

**Published:** 2025-03-03

**Authors:** Kalbiye Konanc, Ergin Ozturk

**Affiliations:** 1 Department of Veterinary, Ordu University, Ulubey Vocational High School, 52850 Ordu, Türkiye; 2 Department of Animal Science, Faculty of Agriculture, Ondokuz MayıS University, 55139 Samsun, Türkiye

**Keywords:** Broiler breeders, İn-ovo injection, Propolis extract, İmmune system, Maternal antibody

## Abstract

This study evaluated the effects of dietary propolis extract supplementation and in-ovo propolis injection on the immune status, blood parameters, vaccine-antibody response, and intestinal microflora of broiler chickens. A total of 600 Ross 308 broiler chicks were used. Breeder diets were supplemented with 400 ppm propolis extract (P) or left unsupplemented (C) during weeks 38–39. Eggs from the control group (C) were divided into four groups, with in-ovo injections of 400 ppm propolis extract (C-iP) or physiological saline (C-iS) on day 18 of incubation. Untreated eggs formed the control (C–C) or propolis-supplemented (P–C) groups. Chicks were fed either a basal diet (C) or a diet supplemented with 400 ppm propolis extract (P), forming six experimental groups: C–C, C-P, P–C, P-P, C-iP, and C-iS, with 10 replicates per group (10 chicks per replicate). Propolis supplementation significantly increased immunoglobulin levels (IgA, IgG, IgM) compared to the control group. Although it positively affected certain blood parameters, no significant differences were found in post-vaccination antibody titers. The C–C group had the highest total antioxidant levels, while total oxidant levels and oxidative stress index were lowest in the P-P group. Despite positive effects on blood parameters and intestinal microflora, no significant improvements in growth performance were observed. Nevertheless, propolis extract shows potential as an immune enhancer for broiler chickens through parental feeding, standard diets, or in-ovo injection.

## Introduction

To regulate and enhance the immune system, scientists have explored various methods, including the use of vaccines to combat external factors and growth-promoting natural products in nutrition, aimed at reducing or eliminating specific pathogens. The immune system of poultry exhibits a complex physiology influenced by numerous factors. These include diet composition (Jha and Mishra 2021), bird age (Song et al. 2021), feeding and energy consumption (Perween et al. 2016), feed additives (Sahin and Ozturk 2018), gut flora (Kim and Lillehoj 2019), genetic potential (Redmond et al. 2010), environmental conditions and stress (Zmrhal et al. 2023). Enhancing poultry immunity significantly impacts both survival rates and fattening performance.

Although maternal antibodies provided through the yolk sac support the chick's immune system in the first days after hatching, they do not offer complete immune protection. Thus, early provision of nutrients that promote immune development can be beneficial (Stefaniak et al. 2020). In-ovo is one of the techniques to administer nutrients to an embryo to aid early take-off, development and stability. In-ovo feeding offers several advantages, such as improved chick quality, higher hatchability and post-hatch body weight, reduced morbidity and mortality, increased meat yield, enhanced gut development and digestive metabolism, altered blood profiles, strengthened immunity, and regulation of gene transcription (Arain et al. 2022).

A variety of materials have been explored for in-ovo applications, with propolis serving as one notable example. Propolis, a natural bee product, has antimicrobial, anti-inflammatory, hepatoprotective, and antioxidative properties, and it enhances biological functions by stimulating the immune system (Anjum et al. 2019). It is rich in biochemical compounds, containing over 300 active components, including phenolic acids, terpenes, cinnamic acid, caffeic acid, flavonoids, and various esters (Ünal and Yıldız 2022). While solvents such as ethyl ether, methanol, water, acetone, chloroform, and dichloromethane can extract and identify propolis compounds, ethanol is considered the most effective solvent for preparing propolis extracts. Primarily due to its ability to dissolve a wide range of bioactive compounds, including flavonoids, phenolic acids, and terpenes, which are responsible for the biological activities of propolis. Compared to other solvents ethanol has superior extraction efficiency, ensuring that a higher concentration of these bioactive components is obtained (Szliszka et al. 2013).

Flavonoids such as Quercetin, Chrysin, Pinocembrin, Galangin, Kaempferol, Apigenin in propolis play a significant role in immune regulation (Ma et al. 2022). They enhance natural killer cell activity against recurrent tumor cells (Takeda et al. 2018), regulate nitric oxide and hydrogen peroxide production by peritoneal macrophages both in vitro and in vivo (Miranda et al. 2015), and increase fungicidal activities of these cells (Mutlu Sariguzel et al. 2016). Due to these properties, propolis and its flavonoids have become topics of intensive research (Sahin and Ozturk 2018; Salatino 2022). Propolis increases immune cell activity by activating and protecting immune cells (Orsatti et al. 2010), regulating cytokines (Zulhendri et al. 2022), boosting antibody production (Al-Hariri 2019), and modulating innate and adaptive immune responses (Wolska et al. 2019). Its multifaceted actions make it a powerful natural supplement for supporting immunity. Propolis has also been reported to enhance antibody production, attributed to its active substances and regulatory effects on innate immune cells, which stimulate antibody synthesis (Al-Hariri 2019).

In-ovo administration has emerged as an alternative to post-hatch vaccination, especially in broilers, due to its lower labor requirements and reduced stress for embryos (Fatemi et al. 2021). Injection into the amniotic fluid surrounding the broiler embryo is performed between 17 and 19 days of incubation (Williams 2011; Omede et al. 2017). Early immune responses in broilers can be initiated through in-ovo injections (Fatemi et al. 2021), and it has been reported that factors encountered during the embryonic period can enhance immune responses later in life (Stefaniak et al. 2020). Furthermore, in-ovo supplementation with additives such as amino acids, vitamins, minerals, carbohydrates, probiotics, plant extracts, and flavonoids has been shown to positively affect the immune system (Kop Bozbay and Ocak 2020; Arain et al. 2022). This method also promotes earlier development of chicks immediately after hatching (Alqhtani et al. 2023).

However, the long-term effects of in-ovo injection of propolis extract on the embryonic immune development of broilers remain largely unexplored. Therefore, this study aimed to evaluate the effects of in-ovo feeding with propolis on the embryonic immune system and assess changes in maternal antibody transfer from breeders to chicks.

## Materials and methods

The study was designed to investigate the effects of propolis extract, both in ovo and through diet supplementation, on immune responses and growth performance in broiler chickens. The experimental design involved several treatment groups, with specific focus on the administration method.

## Propolis extraction

The propolis was stored at −20 ºC until the extraction process. Once hardened, it was thoroughly crushed and ground into a powder using a mortar. To prepare the propolis extract, 30 g of powdered raw propolis was weighed and added to 100 ml of 70% ethanol. The mixture was stored in a cool, dark place for 14 days with the container tightly sealed. During this period, the mixture was stirred 2–3 times daily to ensure maximum contact between the ethanol and the compounds in the propolis (Shalmany and Shivazad, 2006). After the extraction period, the mixture was filtered using Whatman #42 filter paper to remove coarse particles. The filtrate was then transferred to a vacuum evaporator and heated at 50 ºC. After one hour, the complete evaporation of ethanol was confirmed. The resulting extract was stored in amber-colored, screw-capped glass bottles to protect it from light and was prepared for GC–MS analysis (Shalmany and Shivazard 2006). The GC–MS (Gas Chromatography-Mass Spectrometry) analysis results are presented in Table 1.

**Table 1:** Main chemical content of propolis extract (%)

Compounds	(%)
Aldehydes	0.91
Aliphtaic acid and its esters	10.01
Carboxylic acid and its esters	3.27
Flavonoids	16.95
Hydocarbons	1.73
Ketonlar	0.25
Cinnamic acid esters	1.40
Terpenes	1.52

### Determining the propolis extraction dose

A preliminary trial was conducted to determine the optimal propolis extract dose for the study. Once egg production reached 50%, diets were supplemented with four different doses of propolis extract: 100, 200, 400, and 800 ppm. A two-week trial period was initiated. The IgG level in the egg yolk was used as the reference parameter for dose determination. Based on the results, the 400 ppm dose group exhibited the highest maternal antibody (IgG) levels. Consequently, the 400 ppm propolis dose was selected for the study.

## Breeder broiler treatment

One-day-old Ross 308 broiler chicks were housed separately by sex and bred until 18 weeks of age. At 18 weeks, one male was introduced for every 10 females (300 females + 30 males). The birds were provided with adequate hanging feeders, nipple drinkers, perches, and nesting boxes, with wood shavings used as litter material. Maintenance, daily feed amounts (av. 17 g feed intake daily for first day), and nutrient requirements were managed following the recommendations of Ross Anadol 308 guidelines (Table 2). The housing and health protection requirements of the breeders were also carefully adhered to. While the control group continued to receive a basal diet, the propolis group was supplemented with 400 mg/kg propolis extract in their feed. Males and females have same nutritional status and continued for a period of four weeks.

**Table 2:** Feedstuffs and nutrient compositions of the diets; for chicks (1–28 days old)^1^, for pullets (28–154 days old)^2^, for broiler breeders (155 + days old)^3^

Ingredients	%			Nutrient composition	%		
	1	2	3		1	2	3
Corn	547.00	619.00	687.28	Crude protein	19.000	15.000	15.000
SBM*	229.24	109.24	154.16	Eher extract	3.050	3.000	3.370
Wheat bran	72.97	109.15	13.03	Crude Fiber	4.080	4.860	3.550
Wheat meal	60.00	60.00	60.00	Total Ash	5.930	5.200	10.700
SFM**	50.00	100.00	50.00	Total P	0.763	0749	0.583
Limestone powder	11.33	10.14	70.18	Av. Phospho	0.450	0.420	0.350
MCP***	9.41	7.75	6.79	Calcium	1.000	0.900	3.000
Vegetable oil	5.00	1.81	6.57	DL-Methionine	0.423	0.320	0.300
Salmonella inhibit	3.00	3.00	2.00	Lysine	1.010	0.740	0.740
Broiler mix****	3.00	3.00	3.00	Tryptophan	0.236	0.175	0.175
Salt	2.09	2.20	2.20	Threonine	0.724	0.565	0.567
DL-methio. (99%)	1.18	0.47	0.39	İsoleucine	0.798	0.585	0.624
Toxin binder	1.00	1.00	1.00	Histidine	0.520	0.403	0.416
Vitamin D3	1.00	1.00	0.50	Valine	0.901	0.700	0.715
NaHCO_3_	1.00	0.71	1.06	Leucine	1.573	1.244	1.335
Organic minerals	1.00	1.00	1.00	Arginine	1.293	0.969	0.995
Probiotics	1.00	0.50	-	Phenylalanine	0.922	0.692	0.732
Lysine (99%)	0.68	1.67	0.33	Clor	0.167	0.202	0.160
Threonine	0.11	0.27		Sodium	0.160	0.160	0.160
Vitamin E	-	-	0.50	Potassium	0.814	0.634	0.600
				Linoleic acid	1.387	1.325	1.552
				Cholin mg/kg	0.311	0.285	0.286
Total	1000.00	1000.00	1000.00	ME*****	2800	2800	2800

SBM*; Soybean meal (46%CP), SFM**; Sunflower meal (36%CP), MCP (%22.7 Ca)***; Monocalcium fosfat, Broiler mix****; V + M + E = Vitamin + Mineral + Enzyme, ME*****; Metabolisable Energy (Kcal/kg)

### In-ovo feeding

#### Injection of propolis extract into fertile eggs obtained from breeders

After four weeks of feeding with propolis extract, 600 fertile eggs from the control group and the 400 fertile eggs from the propolis-supplemented group (400 mg/kg propolis extract) were collected. The eggs were stored at 14 ºC under 70–75% relative humidity. Prior to incubation, the eggs were preheated to 25 ºC for 24 h. Incubation conditions were set at 37.7 ºC with 60% relative humidity, and the eggs were turned hourly for 18 days during their development. On the 18th day, fertility was assessed, and infertile eggs were discarded by lighting method.

In-ovo injections were administered as follows: 100 fertile eggs from the control group were injected with 400 ppm propolis extract (CiP), while another 100 eggs from the control group were injected with sterile saline (CiS). The eggs were then transferred to hatching machines set at 37.5 ºC with 80% relative humidity for the hatching process.

#### Animal material

After hatching, the chicks were weighed, and a broiler experiment was conducted. The chicks were divided into six experimental groups, each with ten replicates, and ten chicks per replicate.

#### Propolis extract injection

The 400 mg/ml dose, which yielded the best maternal antibody levels when added to the feed and used for parental feeding, was selected for the study. It was deemed appropriate to continue with the same dose for the in-ovo part of the experiment.

130 eggs were selected for each group to guarantee that the minimum required sample size of 100 was met, even if some eggs were lost during the experiment. In-ovo injection of propolis extract was performed on 130 fertile eggs from the control group on the 18th day of incubation. A mixture of 0.4 ml propolis extract and 0.6 ml sterile saline solution (description of the saline mixture is 0.9%) was prepared (Zhai et al. 2011). To maintain the mixture at a temperature similar to the embryo’s environment and to prevent the propolis extract from being affected by high heat, it was kept in a 37 ºC water bath throughout the injection process.

In-ovo feeding (CiP and CiS) was carried out on 260 fertile eggs, which were acclimatized to the temperature of the sterile area used for injection. The eggs were placed under a lighting unit to identify the air spaces and the embryo's position corresponding to the amniotic fluid. The blunt ends of the eggs were cleaned with 70% ethanol, and the designated injection sites were pierced with an 18-gauge needle and a 25-gauge needle. A sterile injection of 1 ml of the propolis extract and saline mixture was then administered into the amniotic fluid of the embryos (Zhai et al. 2011).

To prevent contamination, the needle cavities were sealed with paraffin, which had been kept in a 37 ºC water bath. Eggs that were not injected during the in-ovo procedure were excluded for the duration of the process to ensure equal treatment conditions. All treated eggs were subsequently transferred to the hatching unit.

On the 21st day, the chicks hatched from the eggs were weighed and transferred to pre-prepared compartments for further study.

### Broiler treatment

#### Care and feeding of broilers

Corn-soybean-based diets were prepared for three stages: for days 0–10 (baseline: 24% CP, 3025 ME kcal/kg), for days 11–24 (development: 22% CP, 3150 ME kcal/kg), and for days 25–42 (finishing: 20% CP, 3200 ME kcal/kg) (Table 3). Propolis extracts kept at + 4 ºC were added to the diets at one-week intervals and given to the birds as ad libitum feeding. Broilers were applied the IBDV vaccine on the 14th and 21st days and the NDV vaccine on the 7th and 21st days.

**Table 3:** Feedstuffs and nutrient compositions of the diets used in the broiler experiment: starter (0–10 days old)^1^, grower (11–24 days old)^2^, finisher (25–42 days old)^3^

Ingredients	g/kg			Nutrient composition	%		
	1	2	3		1	2	3
Corn Extra	461.64	500.69	554.46	Crude protein	24.000	22.000	20.000
SBM*	318.88	235.18	173.00	Crude oil	8.720	10.370	10.770
Fullfat soy	150.00	193.05	203.62	Crude cellulose	4.100	4.200	4.230
Vegetable oil	32.72	40.00	40.00	Crude ash	6.750	6.040	5.660
Marble dust	15.30	12.42	12.40	Available phospho	0.500	0.450	0.420
MCP**	12.61	10.23	8.91	Calcium	1.050	0.900	0.850
Broiler mix***	3.00	3.00	3.00	Methionine	0.623	0.537	0.481
Salt	2.88	2.89	2.90	Methionine + Cyctein	1.070	0.950	0.860
DL-meth.99	2.68	2.03	1.70	Lysine	1.437	1.292	1.145
Anticoccidial	0.50	0.50	–––-	Tryptophane	0.317	0.287	0.255
				Arginin	1.782	1.625	1.459
				Linoleic acid	3.872	4.614	4.763
				Cholin (mg/kg)	0.347	0.336	0.323
Total	1000.0	1000.0	1000.0	ME****(Kcal/kg)	3025.0	3150.0	3200.0

SBM*; Soybean meal (%46 CP), MCP (%22.7 Ca)***; Monocalcium fosfat, Broyler mix***; V + M + E = Vitamin + Mineral + Enzyme premix, ME****; Metabolisable Energy (Kcal/kg)

#### Creation of treatment groups (Treatment period, Feeding of animals and health protection)

**Fig. 1:**
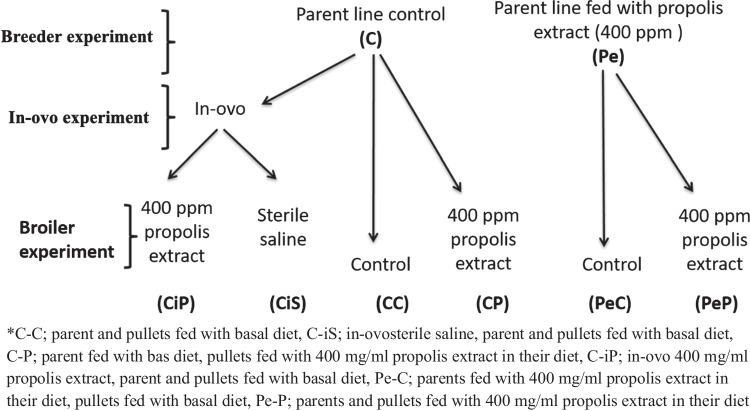
Six experimental groups created for the broiler trial

For the broiler experiment, six groups were formed from the hatched chicks as follows:

1. Control Group (C–C): This group was obtained from breeders that served as the control and continued to be fed without any additives.
2. Propolis-Fed Control Group (C-P): This group was also obtained from control breeders but was fed with a diet supplemented with 400 mg/ml propolis extract.
3. In-Ovo Propolis Group (C-iP): Chicks in this group were obtained from control breeders and received an in-ovo injection of propolis extract on the 18th day of incubation.
4. In-Ovo Saline Group (C-iS): This group was obtained from control breeders and received an in-ovo injection of sterile saline solution on the 18th day of incubation, serving as an additional control for the injection process.
5. Propolis-Supplemented Best Maternal Antibody Transmission Group (Pe-P): Chicks in this group were from breeders that showed the best results in maternal antibody transmission after propolis extract supplementation. These chicks were then fed a diet with the same 400 mg/ml propolis extract dose.
6. Basal Diet Best Maternal Antibody Transmission Group (Pe-C): Chicks in this group were also from breeders with the best results in maternal antibody transmission after propolis supplementation but were continued on a basal diet without additional propolis extract. The experimental groups are presented schematically in Fig. 1.

As shown in Fig. 1, each treatment group had ten replications, with ten chicks per replication regardless of the gender of the chicks. From hatching until the chicks are 42 days old, C–C, C-iS, C-iP and Pe-C groups were fed a diet without added propolis extract. The chicks of the breeder group continued receiving the same propolis extract dose, with 400 mg/ml added to the diet (Pe-P). Another group was created from the control group breeders and continued with a 400 mg/ml propolis extract dose (C-P). The experiment was conducted at Ondokuz Mayıs University Agricultural Application and Research Center. The amount of feed and water the birds could consume was constantly available. Lighting was provided by daylight during the day and fluorescent bulbs at night. The ambient temperature, 32–35 ºC in the first week, was reduced by 2 ºC every week, reaching 20 ºC in the last two weeks.

#### Determination of fattening performance

Live weight, feed consumption, and feed conversion rates were measured in five birds per replication on days 1, 21, and 42. The diets were stored at room temperature and prepared fresh daily before being provided to the birds. The average feed intake of the birds between the two weighing intervals was determined by dividing the total amount of feed consumed by the average body weight gain during the same period. The feed conversion ratios for each period were then calculated. A precision balance with an accuracy of 0.1 g was used for weighing the birds and feed.

#### Detection of maternal antibodies in blood

To measure antibody levels, blood samples (5 ml) were collected from the brachial wing vein of 60 birds, with one bird from each replication and ten birds per group, on days 1, 14, 21, 28, and 42. The selected days for antibody measurements (1, 14, 21, 28, and 42) were aligned with the vaccination schedule. Specifically, the measurements were taken one week after the vaccination to assess the immune response at different stages following the administration of the vaccine.

After collection, the blood samples were centrifuged at + 4 ºC at 1550 × g for 10 min to separate the serum. The collected serums were stored at −80 ºC until analysis. Maternal antibody levels were measured on days 1, 21, and 42, while antibody levels following vaccination were assessed on days 14, 21, and 28, one week after vaccine administration. Quantitative ELISA kits were used to detect maternal antibodies (IgG, IgA, and IgM) in the serum, following the manufacturer's instructions (Bethyl Laboratories, Inc.). The dilutions used in the antibody detection assay were set at 1:20,000 for IgG and 1:10,000 for IgA and IgM. For the analysis of total IgA, IgM, and IgG, anti-chicken immunoglobulins specific to each Ig (1 µl antibody/100 µl coating solution, pH = 9.6) were initially coated onto ELISA plates. The plates were incubated at room temperature for 60 min, followed by three washes with a washing solution (50 mmol Tris-buffered saline containing 0.05% Tween 20, pH = 8). Subsequently, 200 µl of blocking solution was added to each well and incubated at room temperature for 30 min, followed by three washes.

Standards and samples were diluted as specified in the kit instructions, and 100 µl of each was added to the ELISA plate wells. The plates were incubated at room temperature for 60 min and then washed five times. Afterward, 100 µl of diluted peroxidase-conjugated polyclonal anti-chicken Ig was added to each well and incubated at room temperature for another 60 min. The plates were washed five times, and 100 µl of substrate solution was added, followed by reading at 405 nm within 10 min. To stop the reaction, 100 µl of 2 M H₂SO₄ was added to each well.

The plates were read at a primary wavelength of 450 nm using an ELISA microplate reader. Adjustable blank data were transferred to an Excel file. A standard curve was constructed to define the relationship between the concentrations of the standards and their absorbance values for each plate, and the antibody concentrations of the samples were calculated and expressed as micrograms per milliliter or milligrams per milliliter.

#### Anti-NDV and Anti-IBDV antibody detection

To evaluate the antibody levels developed in response to the vaccines, antibody test kits were used to detect Newcastle disease virus (NDV) and infectious bursal disease virus (IBDV) on days 14, 21, and 28. The ELISA plates provided in the assay were pre-coated with inactive viral antigens specific to either NDV or IBDV. The ELISA procedure was performed according to the protocol provided by the manufacturer. Pre-determined working dilutions were used for the samples. The plates were read using an ELISA microplate reader at a primary wavelength of 405 nm and a reference wavelength of 630 nm. Anti-NDV and anti-IBDV antibody levels were determined and calculated by comparing each sample to the positive control. The sample average was calculated by subtracting the absorbance of the negative control from the average absorbance of the sample, and then dividing it by the difference between the average absorbance of the positive control and the average absorbance of the negative control. Since the positive control was prepared at 100 ELISA units (EU), the resulting value was multiplied by 100. The values are expressed in ELISA units. For a more precise assessment of the protective antibody titers, anti-IBDV levels were measured on day 21 in groups vaccinated on day 14. Birds vaccinated on day 7, anti-NDV and anti-IBDV levels were monitored on days 1, 14, 21, 28, and 42, with a particular focus on the results from day 14.

#### Analysis of biochemistry parameters in blood

On the 42nd day, blood was collected in heparin tubes, then centrifuged at 4 °C at 1550 × g for 10 min. The plasma was separated, transferred to Eppendorf tubes, and stored at −80 °C until analysis. Total protein, albumin, triglycerides, aspartate aminotransferase (AST), and alanine aminotransferase (ALT) levels in the serum were measured using an autoanalyzer (AIRONE RA 200) with commercial kits. Total antioxidant (TAL) and total oxidant (TOL) levels were assessed in plasma using the ELISA method. The oxidative stress index was calculated using the TOL/TAL × 100 formula.

#### Determination of intestinal microflora

On the forty-second day, the duodenum, jejunum, and ileum regions of the small intestine were collected as a whole from the birds and transported to the laboratory under aseptic conditions within 4 h. Subsequently, 10 g from each sample were weighed and suspensions were prepared in 90 ml of physiological saline solution. From this stock solution, serial dilutions were made up to 10⁻⁹ in log10 steps, and from these dilutions, two replicate inoculations were made onto the selective agar specific to the bacteria to be determined. A total of 60 intestinal samples (1 from each replicate and 10 from each group) were analyzed for the presence of total aerobic bacteria, *Lactobacillus acidophilus*, *Escherichia coli*, total mesophilic bacteria, and total fungi, following the method described by Sarica et al. (2005).

### Statistical analyzes

To determine the appropriate sample size for this study, a Power and Sample Size calculation was conducted with a significance level (α) of 0.05 and a test power of 95%. Based on this calculation, at least 8 replications per group were deemed necessary to ensure reliable results. However, to further enhance the robustness and reliability of the study, 10 replications per group were utilized.

The assumption of normality for the data was verified using the 1-Sample Kolmogorov–Smirnov test, which confirmed that the data were normally distributed. To compare the means of the groups, a one-way analysis of variance (ANOVA) was conducted. If significant differences were detected between groups (*P < *0.05), post-hoc pairwise comparisons were performed using the Duncan multiple comparison test to identify specific group differences.

Control groups, including the baseline or untreated conditions, were incorporated to provide a clear comparison against experimental conditions and to ensure the validity of the experimental interventions. The control groups were also subject to the same statistical analyses to ensure consistency and accuracy in the results.

All statistical analyses were conducted using SPSS software (Version 21), and a significance level of *P < *0.05 was set for all tests.

## Results

### Maternal antibodies

Based on the serum analysis of the broilers, the C-iP group (in-ovo propolis) showed significant differences compared to both the C–C (control) and C-P (propolis-fed control) groups on day 1. By day 21, the C-iP group also showed significant differences from the C-iS (in-ovo saline) group. On day 42, the C-P group (propolis-fed control) exhibited differences from all other groups, except for the C-iP group. Additionally, the C-iP group had higher IgA levels compared to all other groups. While no significant difference was observed between groups regarding IgG on days 1 and 42, the C–C group exhibited higher values than the Pe-P group on day 21. On day 21, the C-P group had 28.85% higher IgG values than the C–C group (*P* > 0.05).

In our study, the highest IgA values were observed on the 21st and 42nd days when propolis extract was added to the diet. Both dietary supplementation and in-ovo feeding of propolis extract resulted in increased immunoglobulin levels (IgM and IgA) compared to the control group. On day 21, the C–C group exhibited higher IgA values than the Pe-P group. On day 42, the C-iP group showed higher values than all other groups except the Pe-C group, while the C–C group had the lowest IgM values (Table 4).

**Table 4:** Immunoglobulin (IgG mg/ml, IgA and IgM µg/ml) levels in the blood of broiler chickens on days 1, 21 and 42

Treatment	Day	C–C	C-iS	C-P	C-iP	Pe-C	Pe-P	SEM	P
IgG, mg/ml	1	3.71	3.05	3.71	3.38	3.76	3.76	0.187	0.523
	21	4.32^a^	2.99^ab^	3.33^ab^	2.97^ab^	3.01^ab^	2.17^b^	0.195	0.049
	42	4.81	5.49	6.76	5.12	5.07	5.25	0.315	0.551
IgA, µg/ml	1	0.28^b^	0.48^ab^	0.28^b^	0.58^a^	0.41^ab^	0.41^ab^	0.040	0.048
	21	289.00^a^	201.00^b^	305.00^a^	321.00^a^	236.00^ab^	262.00^ab^	12.20	0.037
	42	549.00^c^	539.00^c^	922.00^a^	763.00^b^	535.00^c^	534.00^c^	23.80	0.000
IgM, µg/ml	1	2.88	3.00	2.88	3.40	2.74	2.74	0.147	0.434
	21	495.00^a^	313.00^ab^	359.00^ab^	289.0^ab^	382.00^ab^	261.00^b^	18.60	0.002
	42	406.00^c^	551.00^b^	591.00^b^	739.00^a^	640.00^ab^	539.00^b^	21.40	0.000

^*^C–C; parent and pullets fed with basal diet, C-iS; in-ovo sterile saline, parent and pullets fed with basal diet, C-P; parent fed with bas diet, pullets fed with 400 mg/ml propolis extract in their diet, C-iP; in-ovo 400 mg/ml propolis extract, parent and pullets fed with basal diet, Pe-C; parents fed with 400 mg/ml propolis extract in their diet, pullets fed with basal diet, Pe-P; parents and pullets fed with 400 mg/ml propolis extract in their diet, SEM; standard error of the mean, P; probability

Regarding the immunoglobulin levels in the blood, the C–C group exhibited the highest IgG concentration, measuring 4.32 mg/ml on day 21, while the Pe-P group showed the lowest level at 2.17 mg/ml (Table 4). However, no statistically significant differences were observed in IgG levels between the groups that received propolis supplementation either in ovo or through the diet. These findings suggest that both in-ovo administration and dietary supplementation of propolis extract contribute to an increase in IgG levels, supporting the potential of propolis as an immunomodulatory agent. Despite the absence of significant differences, the trend observed indicates that propolis may play a beneficial role in enhancing immune responses, regardless of the method of application.

### Anti-NDV and Anti-IBDV

Our study evaluated antibody titers in broiler chickens vaccinated against Newcastle disease virus (NDV) on days 7 and 21, with measurements taken one week after vaccination on days 14 and 28. In order to compare the ups and downs in the antibody titers depending on the weeks, the same antibody levels were checked on the 1st, 21st and 42nd days. The chicks have been observed to have antibody titers at the natural level from parental vaccination since hatching (Table 5). Antibodies, which naturally decreased on the 7th day after vaccination, started to rise again when they encountered the antigens in the vaccine. Moreover, there was no difference between the treatments in the titers formed after one week, on the 14th and 28th days (*P* > 0.05), while the highest titer level was observed in the C–C group. However, a regular increase in antibody titers was observed from the 14th day, though a decrease occurred on the 42nd day only in the C-P group, but this difference was statistically insignificant.

**Table 5:** Antibody titers before and after Newcastle (NDV) and Infectious Bursal Disease (IBDV) vaccines in broilers

Treatment	Days	C–C	C-iS	C-P	C-iP	Pe-C	Pe-P	SEM	P
Anti-NDV	1	4773^a^	4593^a^	4773^a^	2751^b^	4804^a^	4804^a^	297.7	0.026
	14	1257	1003	1674	878	762	1011	96.6	0.077
	21	2360^a^	2028^a^	1523^ab^	1490^ab^	1825^ab^	1102^b^	124.0	0.040
	28	4710	3904	4230	3209	2947	3218	235.3	0.201
	42	5367	5234	3645	5069	5470	4519	258.9	0.286
Anti-IBDV	1	4662	4244	4662	4593	4645	4645	114.9	0.543
	14	3011^a^	1485^c^	2327^b^	1945^bc^	1798^bc^	2001^bc^	101.4	0.000
	21	940	785	940	754	803	639	68.1	0.741
	28	566	374	616	468	404	350	40.3	0.225
	42	129	313	137	230	133	298	24.7	0.064

^*^C–C; parent and pullets fed with basal diet, C-iS; in-ovo sterile saline, parent and pullets fed with basal diet, C-P; parent fed with bas diet, pullets fed with 400 mg/ml propolis extract in their diet, C-iP; in-ovo 400 mg/ml propolis extract, parent and pullets fed with basal diet, Pe-C; parents fed with 400 mg/ml propolis extract in their diet, pullets fed with basal diet, Pe-P; parents and pullets fed with 400 mg/ml propolis extract in their diet, SEM; standard error of the mean, P; probability

The data of the 21st and 28th days were evaluated for the antibody titers (Anti-IBDV) against infectious bursal disease vaccinations performed on the 14th and 21st days. A regular decrease in anti-IBDV antibody titers was observed on the 14th, 21st, 28th and 42nd days following vaccinations, and this decrease was accepted as a normal result of the body's physiological response to the vaccines.

In both vaccinations, maternal antibodies, as seen in the Table 5, are higher than the antibodies developed against the vaccines. Antibody titers were very low for each group member after receiving the anti-IBDV vaccine. It should be noted that in the blood samples taken before the 14th day of vaccination, the CC group developed a statistically higher antibody rate of 49.3% than the CiS group. No statistical difference was observed between the groups in developing antibodies for the anti-IBDV vaccine in the weeks after vaccination.

### Blood biochemistry

Among the biochemical parameters, the AST value was the lowest in broilers whose parents were fed control feed, while it was higher by 38.9% in those whose parents were fed propolis extract (*P < *0.05).

Regarding ALT values, the highest levels were observed in broilers whose parents were fed propolis extract, with values of 18.9 IU/L in the C–C group and 17.9 IU/L in the C-P group. The lowest ALT values, 5.0 IU/L, were seen in the Pe-C and Pe-P groups, where the parents were fed control feed (*P < *0.05).

Triglyceride levels were lower in the C-iS group compared to the other groups, except for the C-iP group (*P < *0.05). Furthermore, triglyceride concentrations significantly decreased in the C-iS and C-iP groups, which included in-ovo feeding, compared to the C–C group (*P < *0.05). Our study found that adding propolis extract did not offer a clear advantage compared to the control group, and in-ovo feeding resulted in the lowest antioxidant activity, with concentrations of 0.77 and 0.75 mmol/L (Table 6).

**Table 6:** Biochemical values obtained from blood serum of broilers on day 42

Treatment	C–C	C-iS	C-P	C-iP	Pe-C	Pe-P	SEM	P
ALT, IU/L	18.90^a^	5.90^b^	17.90^a^	6.70^b^	5.00^b^	5.00^b^	0.850	0.000
AST, IU/L	265.00^b^	245.00^b^	265.00^b^	272.00^b^	357.00^a^	368.00^a^	9.100	0.000
Alb, g/dl	1.73	1.72	1.72	1.69	1.69	1.70	0.022	0.993
Total Pro, g/dl	4.25	4.31	4.75	4.50	4.56	4.63	0.060	0.116
Tri, mg/dl	106.10^a^	78.50^c^	94.70^ab^	85.90^bc^	95.0^ab^	95.40^ab^	1.900	0.000
TAL, mmol/L	1.19^a^	0.75^c^	1.03^abc^	0.77^bc^	0.97^abc^	1.11^ab^	0.048	0.037
TOL, µmol/L	5.45^bc^	5.87^ab^	5.92^ab^	6.64^a^	6.17^ab^	4.81^c^	0.150	0.009
OSI	0.46^ cd^	0.57^ab^	0.86^bcd^	0.78^a^	0.64^abc^	0.43^d^	0.044	0.003

^*^C–C; parent and pullets fed with basal diet, C-iS; in-ovo sterile saline, parent and pullets fed with basal diet, C-P; parent fed with bas diet, pullets fed with 400 mg/ml propolis extract in their diet, C-iP; in-ovo 400 mg/ml propolis extract, parent and pullets fed with basal diet, Pe-C; parents fed with 400 mg/ml propolis extract in their diet, pullets fed with basal diet, Pe-P; parents and pullets fed with 400 mg/ml propolis extract in their diet, SEM; standard error of the mean, P; probability

Values: Alanine Aminotransferase (ALT), Aspartate Aminotransferase (AST), Albumin (Alb), Total Protein (Total pro), Triglyceride (Tri), Total Antioxidant Level (TAL), Total Oxidant Level (TOL), Oxidative Stress Index (OSI)

The TAL (Total Antioxidant Level) and TOL (Total Oxidant Level) values, which assess antioxidant and oxidant properties, showed a higher degree of oxidative damage in broilers treated in-ovo (C-iP), with the antioxidant properties being statistically the lowest in both the C-iS and C-iP groups. The lowest oxidative damage was observed in the Pe-P group, where broilers received propolis extract, suggesting that propolis has antioxidant capacity. The C–C group, whose parents and broilers did not receive any additives, had the highest antioxidant levels, while the Pe-P group, supplemented with propolis extract, exhibited the lowest oxidant values.

### Fattening performance of broilers

Live weight (LW), live weight gain (LWG), feed consumption (FC), and feed conversion rates (FCR) were evaluated as fattening performance parameters in broilers. No statistically differences were observed between the C–C, C-P, C-iP, C-iS, Pe-P, and Pe-C groups in terms of LWG on the 1st, 21st, and 42nd days. The feed consumption (FC) of the Pe-C group was higher than that of the other groups at both the 21–42 and 1–42 day intervals. In our study, in-ovo propolis extract feeding did not significantly affect the parameters of LW, LWG, FC, or FCR when compared to the control group. At the end of the study, it was determined that the addition of propolis to the diet or in-ovo feeding (either for the parents or the chicks) did not improve the feed conversion ratio (FCR) compared to the control group (Table 7).

**Table 7:** Live weight (LW), live weight gain (LWG), feed consumption (FC) and feed conversion ratio (FCR) values on 1–21., 21–42. and 1–42. days in broilers

Treatment Days		C–C	C-iS	C-P	C-iP	Pe-C	Pe-P	SEM	P
LW, g	1	48.1	49.9	48.1	47.7	48.3	48.3	0.23	0.076
	21	744.0	803.0	732.0	748.0	793.0	765.0	9.10	0.146
	42	2627.0	2574.0	2572.0	2581.0	2741.0	2628.0	26.50	0.438
LWG, g	1–21	696	754	684	701	745	717	9.10	0.162
	21–42	1883	1770	1840	1833	1948	1859	23.50	0.398
	1–42	2579	2524	2524	2534	2693	2576	26.50	0.435
FC, g/pullet	1–21	1092^c^	1298^a^	1118^c^	1118^c^	1275^ab^	1178^bc^	17.30	0.000
	21–42	3146^abc^	2982^c^	3233^ab^	3005^bc^	3327^a^	2963^c^	35.70	0.007
	1–42	4238^b^	4280^b^	4351^ab^	4122^b^	4603^a^	4141^b^	41.30	0.006
FCR, LWG/FC	1–21	1.57^b^	1.73^a^	1.64^ab^	1.60^b^	1.72^a^	1.64^ab^	0.020	0.027
	21–42	1.68^abc^	1.69^abc^	1.76^a^	1.64^bc^	1.71^ab^	1.60^c^	0.020	0.042
	1–42	1.65^abc^	1.70^ab^	1.73^a^	1.63^bc^	1.71^ab^	1.61^c^	0.010	0.016

^*^C–C; parent and pullets fed with basal diet, C-iS; in-ovo sterile saline, parent and pullets fed with basal diet, C-P; parent fed with bas diet, pullets fed with 400 mg/ml propolis extract in their diet, C-iP; in-ovo 400 mg/ml propolis extract, parent and pullets fed with basal diet, Pe-C; parents fed with 400 mg/ml propolis extract in their diet, pullets fed with basal diet, Pe-P; parents and pullets fed with 400 mg/ml propolis extract in their diet, SEM; standard error of the mean

When considering all the parameters, the fattening performance showed that, although the C-iS group had a 4.6% higher body weight than the C-iP group on day 1, no statistical difference was found. The highest body weight gain from days 1–42 occurred in the Pe-C group. During the 1–42 day period, the C-iP group, which received in-ovo propolis, consumed 11.7% less feed than the control group. Regarding feed conversion ratio from days 1–42, the C-P group achieved the highest value (1.73), while the Pe-P group had the lowest (1.61). It is important to note that the Pe-P group, which showed the highest LWG, also had the highest FC. This suggests that the addition of propolis extract to the feed increases the appetite of broilers, encouraging them to consume more feed.

### Intestinal microflora

In our study, the C–C, C-P, C-iP, C-iS, and Pe-C groups showed similar results in terms of total aerobic microorganism count. However, broiler chickens in the Pe-P group exhibited the highest values for total aerobic microorganisms, significantly higher than the other groups (*P < *0.05). This suggests that aerobic microorganisms may promote fungal growth. Consistent with the total aerobic bacteria findings, the number of Escherichia coli in the aerobic microorganism class was also highest in the Pe-P and Pe-C groups. The results are shown in Table 8.

**Table 8:** Microorganism counts in the small intestine of broiler chickens on day 42 (log10 CFU g-1)

Treatments	C–C	C-iS	C-P	C-iP	Pe-C	Pe-P	SEM	P
Total aerobic	7.00^b^	6.90^b^	6.80^b^	7.00^b^	7.00^b^	7.90^a^	0.057	0.000
Total fungi	5.10^b^	4.90^bc^	4.90^bc^	5.70^a^	4.80^bc^	4.60^c^	0.071	0.000
***E.coli***	6.30^bc^	6.10^c^	6.30^bc^	6.70^b^	7.00^a^	7.00^a^	0.073	0.000
***Lactobacillus*** spp.	7.00^a^	6.40^b^	7.00^a^	6.80^a^	6.90^a^	6.90^a^	0.049	0.001

^*^C–C; parent and pullets fed with basal diet, C-iS; in-ovo sterile saline, parent and pullets fed with basal diet, C-P; parent fed with bas diet, pullets fed with 400 mg/ml propolis extract in their diet, C-iP; in-ovo 400 mg/ml propolis extract, parent and pullets fed with basal diet, Pe-C; parents fed with 400 mg/ml propolis extract in their diet, pullets fed with basal diet, Pe-P; parents and pullets fed with 400 mg/ml propolis extract in their diet, SEM; standard error of the mean

## Discussion

### Maternal antibodies

IgG serves as a critical antibody marker for long-term immunity, playing a vital role in sustained immune defense against pathogens. In our study, the inclusion of propolis significantly enhanced the IgG levels on both the 21st and 42nd days in broilers, suggesting its potential to bolster long-term immunity. This indicates that propolis supplementation may effectively prime the immune system for prolonged protection.

IgM, on the other hand, is recognized as an early-stage antibody, serving as a key indicator of the initial response to antigenic exposure and reflecting a recent activation of antibody production. In our study, despite the absence of any external disease challenges on the 1st day, the IgM levels provided insights into the basal immunological readiness of the broilers. These findings emphasize the dual-phase impact of propolis supplementation, influencing both the early immune response through IgM and the sustained immunity via IgG.

Recent studies have demonstrated that dietary supplementation with propolis can enhance immunoglobulin levels in poultry. For instance, a study found that adding 3 g/kg of propolis to the diet of laying hens resulted in significant increases in serum IgG and IgM levels (Çetin et al. 2010). Similarly, research indicates that broilers fed with propolis exhibit higher serum IgG levels compared to those on a basal diet, suggesting an enhanced antigen-specific antibody response (Al-Kahtani et al. 2022). These findings support the potential of propolis as a natural feed additive to boost humoral immunity in poultry.

Further investigation into the mechanisms driving these immunological changes may provide a deeper understanding of propolis's role as a natural immunomodulator in poultry. IgM levels were elevated in the C-iP and C-iS groups, though not statistically significant. This may be attributed to the intervention occurring prior to hatching in the in-ovo groups. Statistically significant differences in antibody levels were observed between the groups on subsequent days.

IgA, an antibody that accumulates in mucosal areas such as the respiratory and digestive tracts, was found to be lowest on day 1. However, the highest IgA levels on days 21 and 42 were observed in the C-P group, indicating a defensive response triggered by external propolis intake. On day 1, the highest statistical value was recorded in the C-iP group, which received in-ovo propolis, further supporting the role of propolis in stimulating immune defenses.

Zafarnejad et al. (2017) observed significant increases in serum IgG and IgM concentrations in broilers fed with 0.7, 0.8, and 0.9 g/kg propolis, suggesting that high propolis levels modulate humoral immunity. Similarly, Shihab and Ali (2012) reported significant increases in serum IgG and IgM in both broilers and laying hens fed with 3 g propolis/kg. Stefaniak et al. (2020) also supported our findings by demonstrating that in-ovo prebiotics significantly increased IgG levels. These results align with Freitas et al. (2011), who found that propolis application in laying hens boosted IgG and natural IgM production, as well as changes in hematological parameters. It is believed that the increase in IgG levels in propolis-fed birds is related to the stimulation of B lymphocytes via enhanced macrophage activity and cytokine production.

### Anti-NDV and Anti-IBDV

Antibodies against the anti-NDV vaccine showed that the lowest antibody levels were found in the C-iP group prior to vaccination, with this difference being statistically significant. Following vaccination, antibody levels in groups fed with propolis extract were significantly lower compared to the control groups, and these lower antibody titers persisted in blood samples taken at regular intervals until slaughter.

A similar study by Taheri et al. (2005) involving broilers supplemented with propolis extract and administered IBDV vaccines on days 12 and 24 revealed high antibody titers on day 21, followed by a decline by day 42. Additionally, NDV vaccines administered on days 10, 20, and 32 showed similar patterns of antibody titer fluctuations. Nassar et al. (2012) suggested that propolis may act as an immunostimulant in both human and animal vaccines. Ziaran et al. (2005) reported that supplementing broiler diets with propolis extract at various concentrations significantly enhanced antibody production, especially at 70 and 100 mg/kg levels, with effects observed 31 days after NDV vaccination. In contrast, in our study, propolis supplementation, either via diet or in-ovo administration, did not enhance antibodies against anti-NDV. The interaction between the vaccine and propolis could also be a factor. In some cases, strong adjuvants like propolis might alter the vaccine's antigenic presentation or immune response, potentially leading to no observable enhancement in antibody production. This suggests that while propolis can enhance certain aspects of the immune response, its effects on antibody production may vary depending on the specific vaccine and the components used in conjunction with propolis (Fischer et al. 2007).

### Blood biochemistry

Our study found no significant differences between groups in terms of albumin and total protein levels (*P* > 0.05). However, Taha et al. (2019) reported that royal jelly injections (0.5 mL per egg) reduced serum total lipids and globulin while increasing total protein levels compared to other doses (0.25 and 0 mL RJ/egg).

The highest triglyceride levels were observed in the C–C group, while the lowest levels were recorded in the C-iS group. Propolis supplementation did not significantly affect triglyceride levels, whereas in-ovo propolis administration led to a reduction in triglycerides. Taha et al. (2019) similarly reported a decrease in serum triglycerides with royal jelly injections.

Regarding oxidative stress index (OSI) values, propolis supplementation had no positive impact, which contradicts the findings of Seven et al. (2010), who reported alleviation of diet-induced oxidative stress when 1 g/kg propolis and 500 mg/kg vitamin C were added to broiler diets. Akbarian et al. (2016) noted that propolis ethanol extract positively influences oxidative stress only under heat stress conditions. Therefore, it can be concluded that in-ovo propolis feeding resulted in the best OSI values, followed by in-ovo saline, which is more effective than propolis in mitigating oxidative stress. Previous studies have highlighted propolis' potent antioxidant effects among bee products (Diniz et al. 2020).

### Fattening performance

In line with these findings, the highest body weight gain during the 1–21 day period was observed in the C-iS group, with a gain of 754 g. Dang et al. (2022) suggested that in-ovo methionine injections in geese enhanced chest muscle growth, thereby improving live weight (LW) and live weight gain (LWG).

Similarly, Jafari Ahangari et al. (2013) demonstrated that in-ovo royal jelly feeding on day 7 of incubation led to higher LWG, feed consumption (FC), and feed conversion ratio (FCR) at day 21 compared to the control group. Taha et al. (2019) also reported improvements in body weight and daily weight gain in groups receiving 0.5 mL royal jelly/egg injections.

Furthermore, honey supplementation after hatching increases feed intake, FCR, and body weight, while in-ovo honey feeding also boosts these parameters (Memon et al. 2019). The observed low feed consumption in the Pe-P group and high feed consumption in the Pe-C group suggests that the effects of propolis extract on feed consumption may diminish with prolonged feeding (Ziaran et al. 2005). This contrasts with the notion that in-ovo injection, where there is no continuous energy flow from mother to offspring (as in mammals), may be beneficial when nutritional sources are provided externally during incubation (Salmanzadeh et al. 2011).

### Intestinal microorganisms

Bacterial colonization at optimal levels in the digestive system is a key factor that positively influences feed consumption, performance, and overall health in broiler chickens (Kročko et al. 2012). Increasing evidence suggests that intestinal microbiota plays a crucial role in the development and function of the host's immune system (Kim and Lillehoj 2019).

Our study showed no significant differences in *Lactobacillus* spp. counts between groups. However, Memon et al. (2019) observed the highest increase in *Lactobacillus* numbers at in-ovo and post-hatching, with the lowest in the control group. In our study, the Pe-C group, which exhibited high feed consumption and live weight, also had the highest *E. coli* counts. *E. coli* levels were higher in the Pe-P and Pe-C groups compared to the C–C, C-P, C-iP, and C-iS groups, with the C-iP group showing higher counts than the C-iS group. This suggests that feed consumption alterations in the Pe-C group may have influenced the overall microorganism count, but despite these changes, *E. coli* colonization did not reach harmful levels.

On the contrary, Memon et al. (2019) found the highest *E. coli* count in the control group, followed by the in-ovo + honey group, and noted that in-ovo honey application had no significant effect on *E. coli* populations. In our study, broilers receiving propolis had the best results regarding total aerobic and fungal counts, and propolis feeding appeared to reduce the numbers of harmful microorganisms, thus positively contributing to gut health. The lowest *E. coli* count was found in broilers that received in-ovo physiological serum, with in-ovo propolis resulting in significantly lower *E. coli* counts compared to the Pe-C and Pe-P groups. *Lactobacillus* spp., the beneficial intestinal microorganisms, were significantly higher in both C–C and C-P groups, while the in-ovo physiological serum group showed the lowest levels. These results suggest that propolis extract supplementation can enhance *Lactobacillus* populations, though in-ovo administration had no significant effect on bacterial colonization.

## Conclusion

This study assessed the effects of propolis supplementation in breeder diets and in-ovo propolis feeding on the immune system, biochemical parameters, and growth performance of broiler chicks. The addition of 400 mg of propolis extract to the breeder diet did not significantly improve the chicks' immune system, but it positively impacted their growth performance, particularly feed efficiency (*P < *0.016). In-ovo propolis feeding increased body weight gain (BWG) on day 42 (*P < *0.003), reduced oxidative stress (*P < *0.003), and improved the feed conversion ratio (FCR) (*P < *0.016). However, no effect was observed on anti-NDV and anti-IBDV antibody levels.

Both in-ovo and feed-added propolis increased immunoglobulin levels, supporting the immune system by 5.4–40.5% compared to the control group. The in-ovo feeding of propolis caused an increase in fungal counts on day 42 compared to the feed addition group. Overall, propolis extract demonstrated potential as an immune enhancer, and in-ovo feeding may offer a more advantageous method than post-hatching supplementation.

## Data Availability

The datasets generated during and/or analysed during the current study are available in the [Figshare] repository, [10.6084/m9.figshare.24941931.v1].
